# Effects of different obesogenic diets on joint integrity, inflammation and intermediate monocyte levels in a rat groove model of osteoarthritis

**DOI:** 10.3389/fphys.2023.1211972

**Published:** 2023-07-13

**Authors:** K. Warmink, J. L. Rios, D. R. van Valkengoed, P. Vinod, N. M. Korthagen, H. Weinans

**Affiliations:** ^1^ Department of Orthopedics, University Medical Center Utrecht (UMCU), Utrecht, Netherlands; ^2^ Department of Equine Sciences, Utrecht University, Utrecht, Netherlands; ^3^ Department of Biomechanical Engineering, TU Delft, Delft, Netherlands

**Keywords:** osteoarthritis, obesity, inflammation, monocyte, macrophage, metabolic syndrome

## Abstract

**Introduction:** Obesogenic diets aggravate osteoarthritis (OA) by inducing low-grade systemic inflammation, and diet composition may affect OA severity. Here, we investigated the effect of diet on joint damage and inflammation in an OA rat model.

**Methods:** Wistar-Han rats (*n* = 24) were fed a chow, a high-fat (HF) diet, or a high-fat/high-sucrose (HFS) for 24 weeks. OA was induced unilaterally 12 weeks after the diet onset by groove surgery, and compared to sham surgery or no surgical intervention (contralateral limb). Knee OA severity was determined by OARSI histopathology scoring system. At several timepoints monocyte populations were measured using flow cytometry, and joint macrophage response was determined via CD68 immunohistochemistry staining.

**Results:** Groove surgery combined with HF or HFS diet resulted in higher OARSI scores, and both HF and HFS diet showed increased circulating intermediate monocytes compared to chow fed rats. Additionally, in the HFS group, minimal damage by sham surgery resulted in an increased OARSI score. HFS diet resulted in the largest metabolic dysregulation, synovial inflammation and increased CD68 staining in tibia epiphysis bone marrow.

**Conclusion:** Obesogenic diets resulted in aggravated OA development, even with very minimal joint damage when combined with the sucrose/fat-rich diet. We hypothesize that diet-induced low-grade inflammation primes monocytes and macrophages in the blood, bone marrow, and synovium, resulting in joint damage when triggered by groove OA inducing surgery. When the metabolic dysregulation is larger, as observed here for the HFS diet, the surgical trigger required to induce joint damage may be smaller, or even redundant.

## Introduction

Osteoarthritis (OA) is a major cause of disability worldwide, affecting over 40 million individuals in Europe (WHO. WHO Scientific Group, 2003). Obesity, and consequently obesity-associated metabolic syndrome, is a significant risk factor for OA, impacting weight-bearing joints like the hip and knee, as well as non-weight-bearing joints such as the hand (Reyes et al., 2016; Gu et al., 2019; Long et al., 2019). This association provides strong evidence for a systemic component in obesity-related OA, as excessive mechanical loading alone cannot account for the increased risk in non-weight-bearing joints. Moreover, joint pain at multiple sites is observed in 84% of the OA population, suggesting OA is often not a single joint disease and can act systemically (Badley et al., 2020).

In obesity, stressed white adipose tissue produces inflammatory mediators like leptin and other adipokines, which, upon entering circulation, induce low-grade chronic inflammation (Francisco et al., 2018). In addition, unhealthy diets rich in saturated fatty acids and sugars exert direct, predominantly negative, effects on immune cells. For example, saturated fatty acids increase the production of tumor necrosis factor (TNF) and interleukin-1β (IL-1β) in macrophages and enhance activation and differentiation of pro-inflammatory T helper (Th)17 and Th1 phenotypes (Snodgrass et al., 2013; Radzikowska et al., 2019). Dietary sucrose activates nuclear factor-κB (NF-κB) signaling in macrophages and other immune cells, leading to increased production of pro-inflammatory cytokines (Shomali et al., 2021). Additionally, high levels of dietary saturated fatty acids and sugars contribute to the development of obesity-induced metabolic syndrome and gut barrier dysfunction, also known as leaky-gut syndrome (Binienda et al., 2020). Increased gut permeability allows the entry of bacteria and their products into the bloodstream, further contributing to systemic inflammation (Natividad and Verdu, 2013; Mu et al., 2017).

Upon entering the bloodstream, inflammatory mediators released by adipose tissue and the gut activate circulating immune cells, including monocytes. Monocytes, categorized into classical, non-classical, and intermediate subsets, play a crucial role in the innate immune system (Ziegler-Heitbrock et al., 2010; Zawada et al., 2011). Classical monocytes are primed for innate immune responses and phagocytosis, whereas non-classical monocytes are involved in adhesion and complement activation (Wong et al., 2011; Kapellos et al., 2019). Intermediate monocytes have a differentiation state in between classical and non-classical monocytes and are associated with the secretion of inflammatory cytokines (Wong et al., 2011; Wong et al., 2012; Patel et al., 2017). Higher levels of intermediate monocytes have been observed in individuals with obesity, rheumatoid arthritis, and other inflammatory diseases (Poitou et al., 2011; Rossol et al., 2012; Wong et al., 2012; Ruiz-Limon et al., 2019). In patients with knee OA elevated levels of intermediate monocytes in the synovial fluid correlated with worse Knee injury and Osteoarthritis Outcome Score (KOOS) and Western Ontario and McMaster Universities Osteoarthritis Index (WOMAC) function scores (Gomez-Aristizabal et al., 2019). Furthermore, *in vitro* human intermediate monocytes have shown an increased capacity to form osteoclasts with a high bone resorption capacity (Sprangers et al., 2016). Therefore, increased inflammatory monocytes populations could contribute to the development of OA, via 1) direct secretion of inflammatory mediators in the synovium, 2) via differentiation into pro-inflammatory macrophages in the synovium, and/or 3) via differentiation into (over)active osteoclasts that can lead to (subchondral) bone related alterations and deformities of the joint.

Animal studies have demonstrated that both joint destabilization, and diet-induced obesity can result in alterations in peripheral monocyte subsets and increased joint degeneration (Takahashi et al., 2003; Rios et al., 2019; Utomo et al., 2021). Additionally, in a rat model of OA induced by combined high-fat diet and surgical damage, elevated levels of macrophage activation and joint degeneration have been observed (de Visser et al., 2018a; de Visser et al., 2018b), both on folate single-photon emission computed tomography (SPECT/CT) macrophage imaging and immunohistochemistry staining. This suggest an association between diet-induced inflammation in monocytes and macrophages, and the development of OA. Moreover, numerous studies have demonstrated that diet-induced obesity exacerbates OA in post-traumatic OA models (de Visser et al., 2018b; Sudirman et al., 2020; Tang et al., 2020; Cao et al., 2022). However, most studies use joint destabilization methods that cause significant damage to induce OA, while it may be clinically relevant to investigate the effects of a long-term obesogenic diet combined with a mild or slowly developing joint damage for a subgroup of patients.

In the current study, we aimed to investigate the link between obesity, inflammation, and OA by examining systemic monocyte and local macrophage responses in the rat groove model, by combining two different obesogenic diets with mild cartilage damage. Various obesogenic diets have been used in preclinical studies to investigate the relationship between obesity and OA in animal models (Griffin et al., 2012; Collins et al., 2018; Donovan et al., 2018; Kozijn et al., 2018; Rios et al., 2019). The high-fat (HF) diet groove rat model was established to mimic metabolic OA, characterized by low-grade systemic inflammation and gradual joint degeneration (de Visser et al., 2017; de Visser et al., 2018b; de Visser et al., 2019; Warmink et al., 2022). In this model, rats were subjected to a 24-week HF diet, followed by surgical OA induction at 12 weeks, modeling the development of OA in patients who have been exposed to an obesogenic diet for an extended period before experiencing joint injury or other triggers leading to OA (de Visser et al., 2017; de Visser et al., 2018b). This approach might better mirror the progression of OA in patients with metabolic OA, where an unhealthy diet is likely to be established long before the onset of OA symptoms (Braddon et al., 1986; Pachucki, 2012; Thomas et al., 2018). Moreover, the composition of the diet seems to affect the development of OA, with certain studies suggesting that high dietary sucrose intake leads to more joint degradation (Collins et al., 2018; Donovan et al., 2018; Rios et al., 2019). Thus, in addition to the HF diet typically used in the groove OA model, we sought to investigate the effects of a high-fat/high-sucrose (HFS) diet, with a sucrose component reflective of unhealthy dietary habits observed in humans (Johnson and Wardle, 2014; de Moura et al., 2021; Janoschek et al., 2023). By comparing chow, HF and HFS diet, we aimed to evaluate the impact of these distinct dietary compositions on OA pathogenesis within the context of the groove OA model, which represents metabolic OA patients with slowly progressing joint degeneration. We hypothesize that an obesogenic diet, particularly the HFS diet, will result in greater inflammatory monocyte and macrophage responses compared to a chow diet and, when combined with mild cartilage damage, will lead to more severe OA due to pre-existing inflammation and metabolic dysregulation.

## Methods

### Animals and OA model

In total 24 (12 week old, male) Wistar-Han rats [Crl:WI(Han) Charles-River, Sulzfeld, Germany] were fed *ad libitum* with either a regular chow diet (*n* = 8 rats; “rat and mouse breeder and grower 801730”; 22 kcal% protein, 9 kcal% from fat, 69 kcal% from carbohydrates of which 8.4% sucrose, SDS Diets, Essex, United Kingdom), a high-fat diet (*n* = 8; customized diet “EF D12451 without sucrose”; 20 kcal% protein, 60 kcal% fat of which 100% pork lard, 20 kcal% carbohydrates of which 0% sucrose, Sniff Bio-Services, Soest, NL), or a high-fat/high-sucrose diet (*n* = 8; customized diet “EF HS D12451 mod.”; 20 kcal% protein, 40 kcal% fat of which 94% pork lard, 40 kcal% carbohydrates of which 83% sucrose**,** Sniff Bio-Services, Soest, NL), for a period of 24 weeks. Diet allocation was randomized by weight at baseline. Twelve weeks after the diet onset, cartilage damage was made to surgically induce OA by unilateral placement of grooves on the femoral condyles in the right knee joint in 4 rats in the chow group and in 5 rats in the HF and HFS groups. More animals received groove surgery in the HF and HFS groups because the variation in obese grooved animals was found to be larger than in the chow and sham animals (de Visser et al., 2018b); the supporting *a priori* power analysis is described in the statistical section. The remaining rats of each diet group received sham surgery in their right joint as a control. During the groove surgery the knee joint cavity was opened with a small longitudinal incision through the patellar tendon. Next, local cartilage damage was induced by making five longitudinal grooves on the femoral condyles and trochlea as described previously (de Visser et al., 2017). The groove tool was adapted from a fine dura retractor surgical tool (FD376R Aesculap Surgical Instruments, B. Braun AG, Melsungen, Germany), where the tip of the tool was shortened to 150 µm. During sham surgery the same procedure was followed as for the groove surgery, piercing the groove tool though the incision in the patella, infrapatellar fat pad and synovium, but no grooves were placed on the cartilage. Animals were allocated to either groove or sham surgery using digitally generated random numbers. The left knee joints of all animals were preserved (no surgical intervention) and served as a non-surgery control. Although it is well-documented that OA in one knee joint can lead to abnormal biomechanics in other joints in humans and dogs, and in DMM mouse models (Mundermann et al., 2005; Bockstahler et al., 2009; Metcalfe et al., 2013; Fouasson-Chailloux et al., 2021; Alves et al., 2022), our relatively mild OA model with unilateral groove surgery did not show significant differences in weight bearing between the left and right joints when untreated, as shown previously (Warmink et al., 2023). All surgeries were performed under general anesthesia (isoflurane–induced at 4%, maintained at 2.5%), and analgesia (0.01 mg/kg buprenorphine, injected subcutaneously prior to surgery, and 6–8 h after first injection). In addition to the weekly monitoring, animals were daily weighed and checked for signs of inflammation, pain or sensitivity to touch of the operated limb, and willingness to put weight on the operated limb, for a minimum of 2 days after surgery. Groove surgery seemed to result in minimal discomfort (no signs of redness, swelling, sensitivity to touch or unloading after groove surgery). This might be explained by the nature of the surgery, where the joint is minimally opened via a longitudinal incision through the ligamentum patellae, where the tip of the groove tool is inserted, while the rest of the joint cavity remains closed. At the end of week 24 in the experimental protocol, animals were euthanized by aorta puncture under general anesthesia (isoflurane, 4%), and tissues of interest were harvested for further evaluation. Rats were housed per two in open polycarbonate cages (Type IV) provided with nesting material, nest box and solid wood block, under a 12:12 light-dark cycle, with water *ad libitum*. No animals were excluded during the experiment. Humane endpoints were established as *a priori* exclusion criteria, these were: bacterial infection or signs of severe discomfort (joint unloading and/or >15% weight loss). Study protocol was approved by the Utrecht University Medical Ethical Committee for animal studies (license AVD115002016490) and was in compliance with European Community specifications regarding the use of laboratory animals. Study protocol was not pre-registered in an online animal study registry.

### *In vivo* measurements

Body mass was measured weekly. At week 0, 12 and 24, animals were fasted for 16-h and blood was obtained from the lateral tail vein and collected in both lithium heparin plasma, and clot activator serum tubes (BD Microtainer, BD, Franklin Lanes, United States). During blood collection rats were restricted using a cloth towel, and no anesthesia was used. Samples were placed on wet ice, then centrifuged (15 min, 3000 g) within 30 min after collection. Plasma and serum aliquots were stored at −80 C until analysis. Heparin plasma was used to determine glucose and triglyceride levels (University Veterinary Diagnostic Laboratory of the Utrecht University). Serum insulin levels were determined by ELISA (EZRMI-13K, Millipore, Amsterdam, Netherlands). Insulin resistance index (HOMA-IR) was calculated according to the equation proposed by Matthews et al., (1985), where HOMA-IR = glucose (mmol/L) * insulin (mU/L)/22.5. In addition, a glucose tolerance test was performed at week 24, after 16 h of fasting, where rats received 2 g/kg of glucose by oral gavage. Blood was collected in a serum tube at 0, 15, 30, 60, 90 and 120 min after gavage from the tail vein for insulin measurement using ELISA (EZRMI-13K, Millipore, Amsterdam, Netherlands). Blood glucose was measured immediately with a blood glucose meter (OneTouch Verio and Blood Glucose Monitoring System, Lifescan, Switzerland). Composite insulin sensitivity index (CISI) was determined using the glucose (mMol/L) and insulin (pmol/L) values from the glucose tolerance test, where CISI = 1,000/√(t = 0 insulin*t = 0 glucose)*(average all time points insulin*average all time points glucose) (Matsuda and DeFronzo, 1999). Serum cytokines and chemokines of the 24 weeks timepoint were measured using a rat 27 multiplex assay (Eotaxin, EGF, Fractalkine, IL-1α, IL-1β, IL-2, IL-4, IL-5, IL-6, IL-10, IL-12(p70), IL-13, IL-17A, IL-18, IP-10/CXCL10, GRO/KC, IFN-γ, TNF-α, G-CSF, GM-CSF, MCP-1, Leptin, LIX, MIP-1α, MIP-2, RANTES, VEGF; MILLIPLEX RECYMAG65K27PMX, Millipore, Darmstadt, Germany; assay was performed at the MultiPlex Core Facility of the University Medical Center Utrecht, the Netherlands). EGF was not included in the final analysis as the majority of values was out of range.

### Monocyte flow cytometry

In weeks 1, 6, 11, 17 and 23 in the study, approximately 0.5 mL of blood was obtained from the tail vein and collected in EDTA tubes (BD Microtainer, BD, Franklin Lanes, United States). Following erythrocyte lysis, cells were washed twice in FACS buffer (1% BSA and 0.1% Na-azide in PBS, all from Sigma-Aldrich, Saint Louis, United States of America). Cells were transferred to a V-bottom polypropylene 96-well plate and incubated at 4°C for 30 min in FACS buffer with 1% rabbit serum, 1% Fc-receptor blocker (Innovex Biosciences, Essex, CA, United States) and the fluorescent antibodies shown in Table 1. Pooled portions of samples were used for unstained, single-stained and fluorescence minus one (FMO) controls. After incubation cells were washed and resuspended in FACS buffer containing 0.95 uM DAPI for live/dead staining. Data acquisition was performed on a 4-laser Cytoflex S (Beckman Coulter, Indianapolis, United States), with plate reader using the CytExpert software. Populations of interest were gated according to the gating strategy as depicted in Supplementary Figure S1, to obtain the monocyte population frequencies. In short, debris, doublets and dead cells were excluded, then cells with lower granularity were selected on a forward/side scatter plot (Strauss-Ayali et al., 2007). From these cells the CD172a^+^ monocyte population was selected, and low, intermediate and high CD43-expressing monocyte populations were determined (Melgert et al., 2012; Barnett-Vanes et al., 2016). In addition, the CD11b/c^+^ percentage of the overall CD172a^+^ monocyte population was determined.

**TABLE 1:** Flow cytometry antibody panel used to stain monocyte populations.

Marker	Fluorochrome	Dilution	Clone	Manufacturer	References
Anti-rat CD11b/c	AF488	1:400	OX-42	Biolegend	201812
Anti-rat CD43	PE-Cy7	1:300	W3/13	Biolegend	202816
Anti-rat CD172a	PE	1:200	OX-41	Biolegend	204706

### Micro computed tomography

In week 24 micro computed tomography (micro-CT) scans were made using a Quantum FX micro-CT scanner (Caliper Life Sciences, PerkinElmer, Waltham, United States). During the scans rats were under general anesthesia (2.5% isoflurane) and both hind limbs were positioned in extension. Knee joints were scanned at an isotropic voxel size of 42 μm, voltage of 90 kV, current of 180 μA and a field of view of 21 mm. ImageJ software (ImageJ, 1.47v, NIH, Bethesda, United States of America) was used for analyses. In serial 2D images in coronal orientation the osteophyte diameter was measured. Additionally, the bone was segmented using a local threshold algorithm (ImageJ, Bernsen algorithm using radius 5) to evaluate tibial subchondral plate thickness (μm), trabecular bone thickness (μm) and volume fraction using the BoneJ plugin in ImageJ (Domander et al., 2021). The trabecular bone volume fraction (BV/TV) was calculated by the ratio of trabecular bone volume (BV, in mm^3^) and total endocortical tissue volume (TV, in mm^3^). Regions of interest were manually drawn in coronal orientation in 90 slides, starting at the back of the joint at the point where the medial and lateral compartment of the tibial epiphysis are first connected, moving to the front of the joint.

### Histology

Knee and hip joints were harvested and fixated in 4% neutral buffered formaldehyde for 1 week and then decalcified in 0.5M EDTA (set to pH7.0 with NaOH) for 6 weeks. Every week the samples were re-fixated in 4% neutral buffered formaldehyde for 24 h. The decalcified tissue was then dehydrated in a series of ethanol (70%–100% ethanol), cleared in xylene, and paraffin infiltrated. Knee joints were paraffin embedded in a 90°–100° angle with the patella facing down, then 10 coronal 5 µm sections were made at 200 µm intervals throughout the knee joint. Sections of the knee joints were stained with Weigert’s Hematoxylin, Fast Green and Safranin-O, and the joint degeneration was then evaluated using the OARSI histopathology score for rats, as described in Gerwin et al., (2010). In short, the three most severely affected sections, determined by the area that contained the most severe cartilage lesions, from each joint were scored. All sections were scored in a blinded and randomized order, by an observer with 2 years of experience. The total OARSI score is presented as the sum of the following subscores: cartilage degeneration (0–60), calcified cartilage and subchondral bone damage (0–5), osteophyte size (0–4) and synovial membrane inflammation (0–4). The cartilage degeneration score comprised the sum of four cartilage compartments (tibia and femur, medial and lateral), where each compartment was scored on a scale of 0–15. The surgical applied grooves were not considered during the scoring, only the cartilage adjacent to the grooves was included.

Immunohistochemistry staining for CD68 was performed to look at the presence of monocytes and macrophages in knee and hip joints. Slides were blocked for endogenous peroxidase using 0.3% H_2_O_2_ for 10 min at room temperature. Antigen retrieval was performed using 0.1% pepsin (Sigma, Saint Louis, United States) in 0.02M HCl solution for 30 min at 37°C and all slides were blocked in 5% PBS-BSA for 30 min at room temperature. Primary antibody incubation was done overnight at 4°C with 0.75 μg/mL CD68 mouse monoclonal antibody (ab31630, Abcam, Cambridge, United Kingdom) or with 0.75 μg/mL normal mouse IgG1 as negative control (sc3877, Santa Cruz, Dallas, TX, United States). The next day slides were incubated with anti-mouse HRP (Envision, Dako, CA, United States) for 30 min at room temperature and subsequently incubated with liquid DAB+ 2-component system (Agilent, Santa Clara, CA, United States) for 5 min. Sections were counterstained with hematoxylin, dehydrated in a series of alcohols, cleared in xylene and coverslipped with Eukitt mounting medium (Sigma-Aldrich, Saint Louis, United States). For each structure of interest (bone marrow, synovial lining and periosteum) three representative images per joint were captured at a ×10 magnification. CD68 staining was quantified in ImageJ (1.47v, Bethesda, MD, United States), the region of interest (ROI) was manually drawn in each image, as depicted in Supplementary Figure S2. The region of interest included all areas with the structure of interest (bone marrow, synovial lining or periosteum) that was observed in the image. Images were (colour) deconvoluted with vector set to “H DAB” (Ruifrok and Johnston, 2001). The brown colour-separated image was used to determine the percentage area of positive staining within the selected ROI, using threshold and measure functions. Threshold was determined for each tissue of interest (bone marrow, synovial lining and periosteum), the same threshold was used for every image of the respective tissue.

### Statistics

**FIGURE 1:**
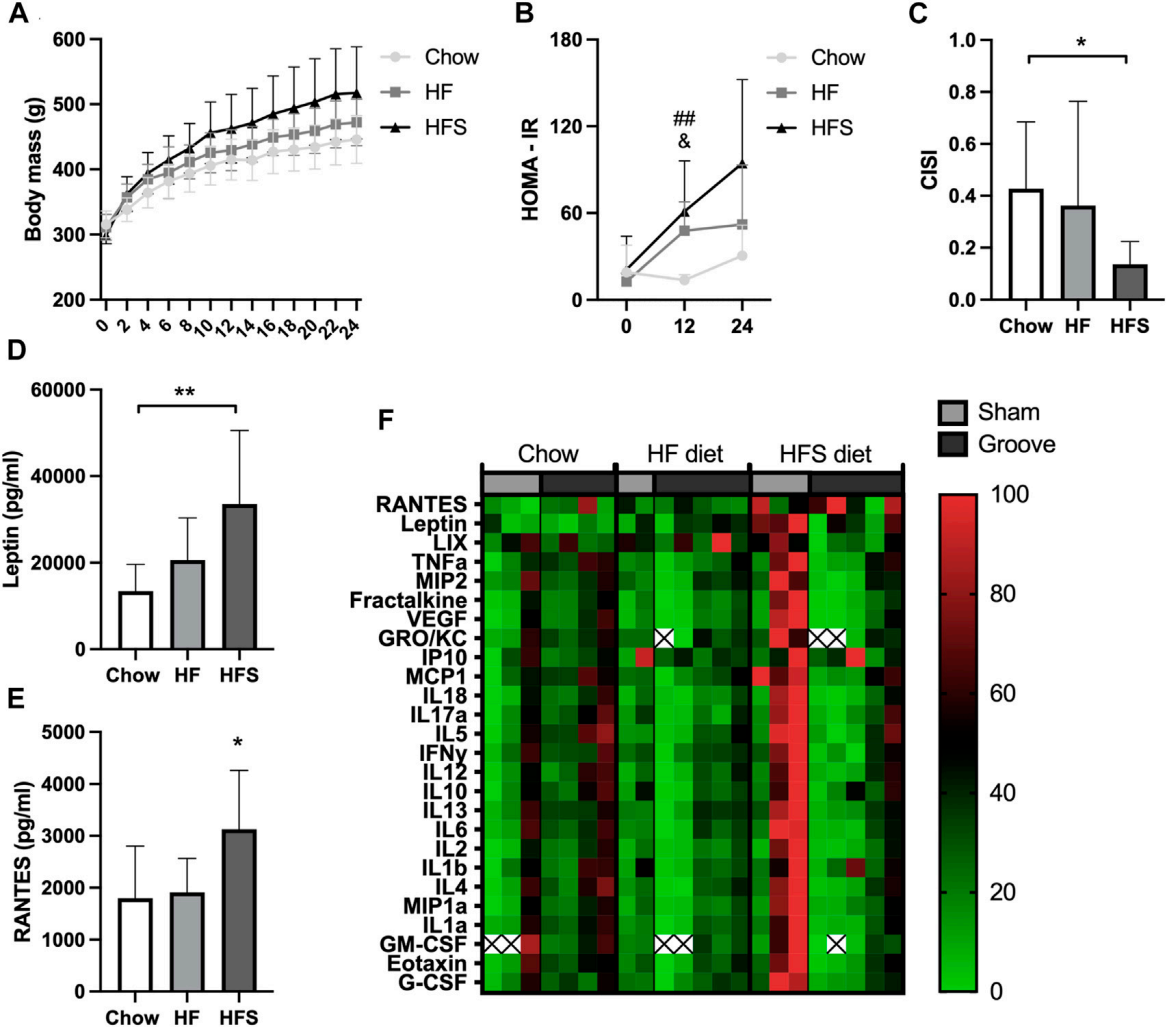
Effects of diet on systemic metabolic and inflammatory markers. **(A)** Body mass of the three diet groups over time. **(B)** HOMA-IR of the three diet groups measured at week 0, 12 and 24. #: indicates significance between HF and chow, &: indicates difference between HFS and chow. **(C)** CISI determined via glucose tolerance test at week 24. **(D)** Serum leptin and **(E)** RANTES levels, determined from week 24 serum. **(F)** Normalized cytokine (highest value set to 100, lowest value set to 0) multiplex values, each column represents one animal. Two animals (one chow, one HF) were excluded from cytokine analysis due to low sample quality (hemolysis) resulting in many out of range values. The remainder out of range values are indicated with a cross.

**FIGURE 2:**
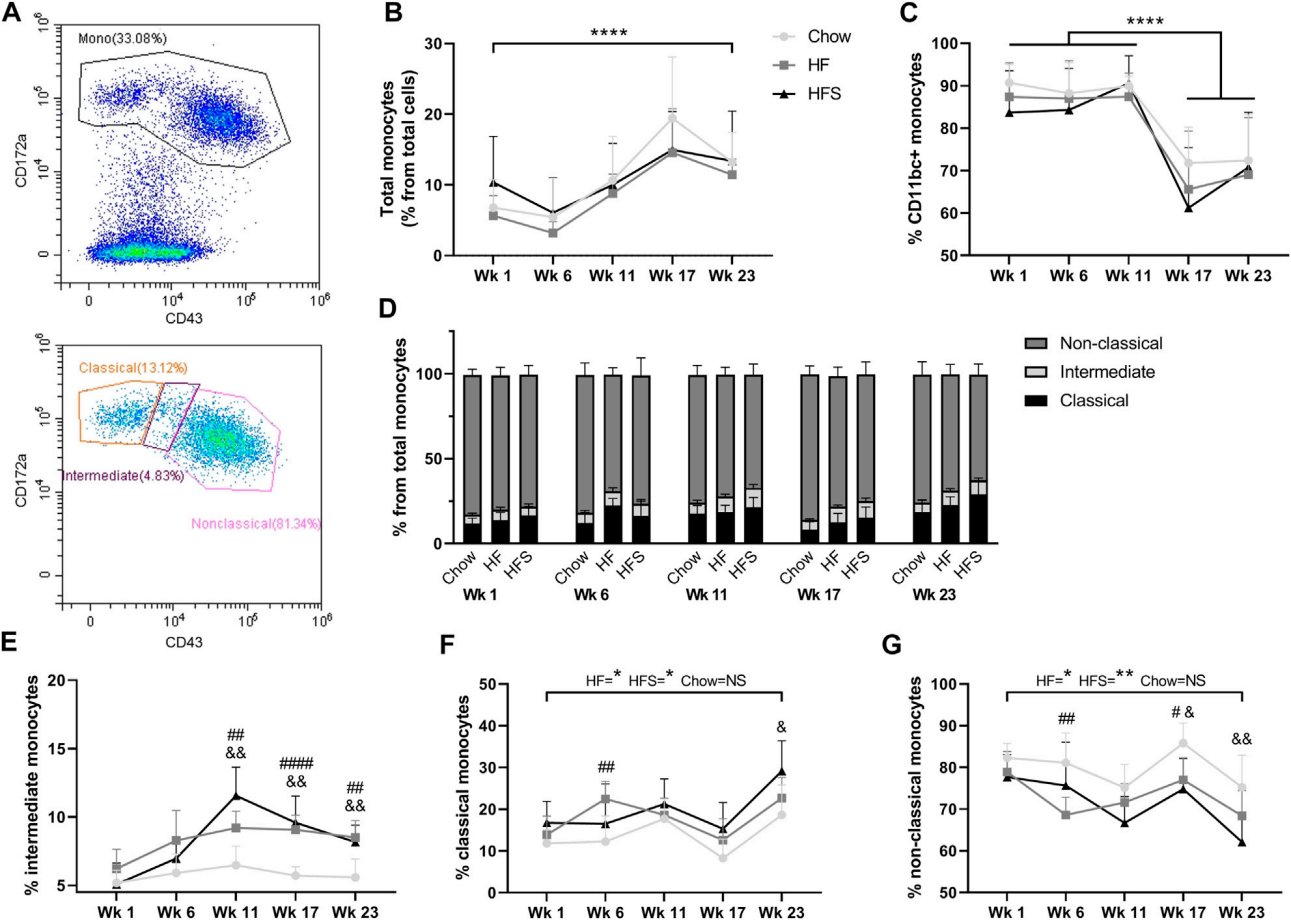
Effects of diet on circulating monocyte populations. **(A)** representative image of monocyte gating, using CD43 and CD172a. **(B)** Percentage of monocytes from total leukocytes show for the three diets at different time points. **(C)** Percentage of CD11bc + monocytes from total monocyte population. **(D)** Non-classical, intermediate and classical monocyte populations show in stacked bars as a percentage from total monocyte population (summarizing figures E–G). Monocyte subpopulations shown separately as **(E)** percentage intermediate monocytes, **(F)** percentage classical monocytes and **(G)** percentage non-classical monocytes. *: indicates a significant difference between time points. #: indicates significance between HF and chow at one time point, &: indicates significance between HFS and chow at one time point.

**FIGURE 3:**
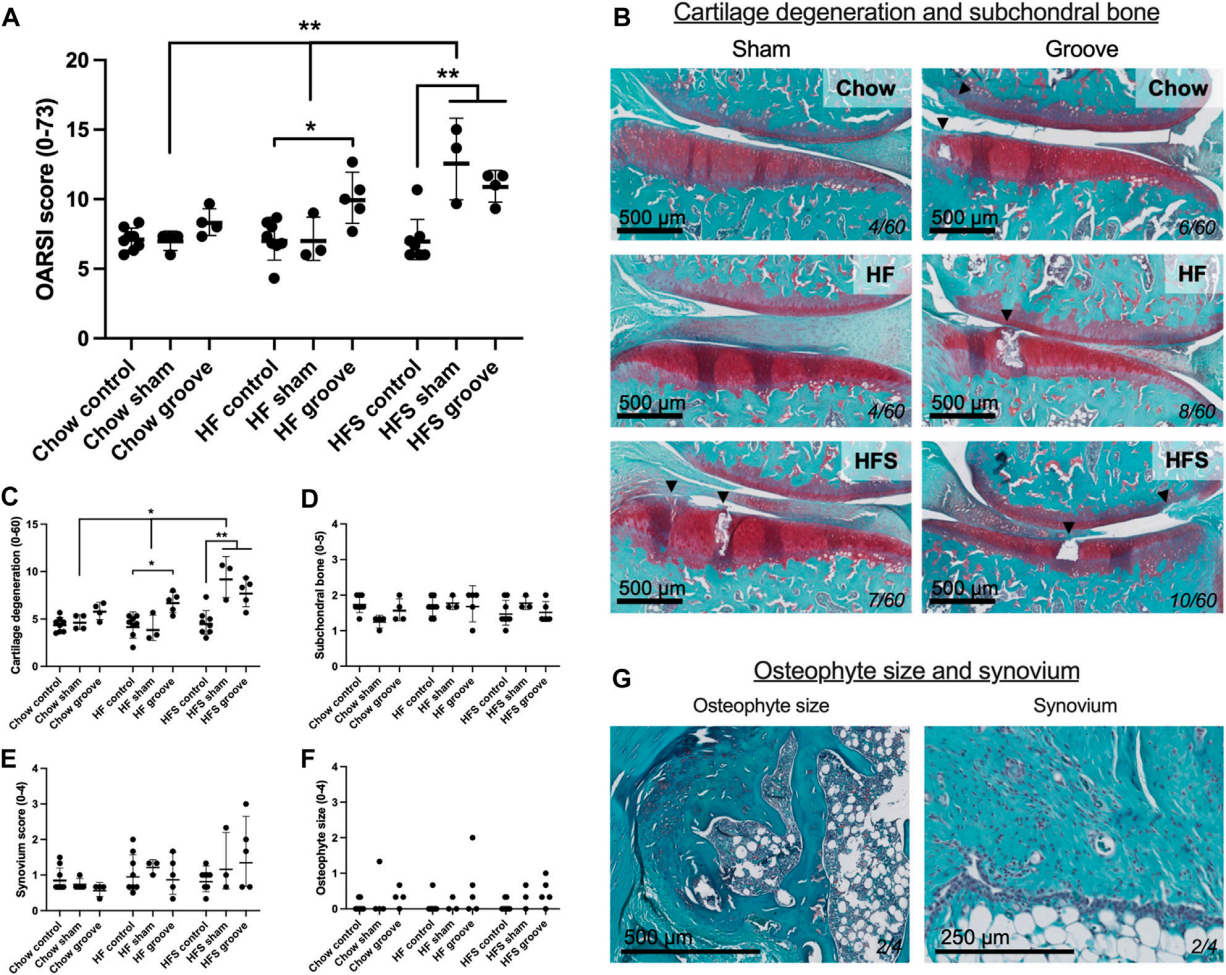
Effects of diet on joint degeneration. **(A)** Total OARSI score measured on Safranin-O stained tissue sections. **(B)** Representative histological images of sham and grooved joints in the chow, HF and HFS groups. Cartilage and subchondral bone are visible, arrowheads indicate damaged cartilage areas, cartilage damage subscore of the section is shown in bottom-right corner. OARSI subscores of **(C)** cartilage degenerations, **(D)** subchondral bone damage, **(E)** synovial inflammation and **(F)** osteophyte size. **(G)** Representative images of a medium osteophyte and synovium, subscores of the section are shown in bottom-right corner. Please note that all statistics on scores were performed on the log-transformed values in order to meet statistical requirements, therefore graph represents geometric mean and geometric SD.

**FIGURE 4:**
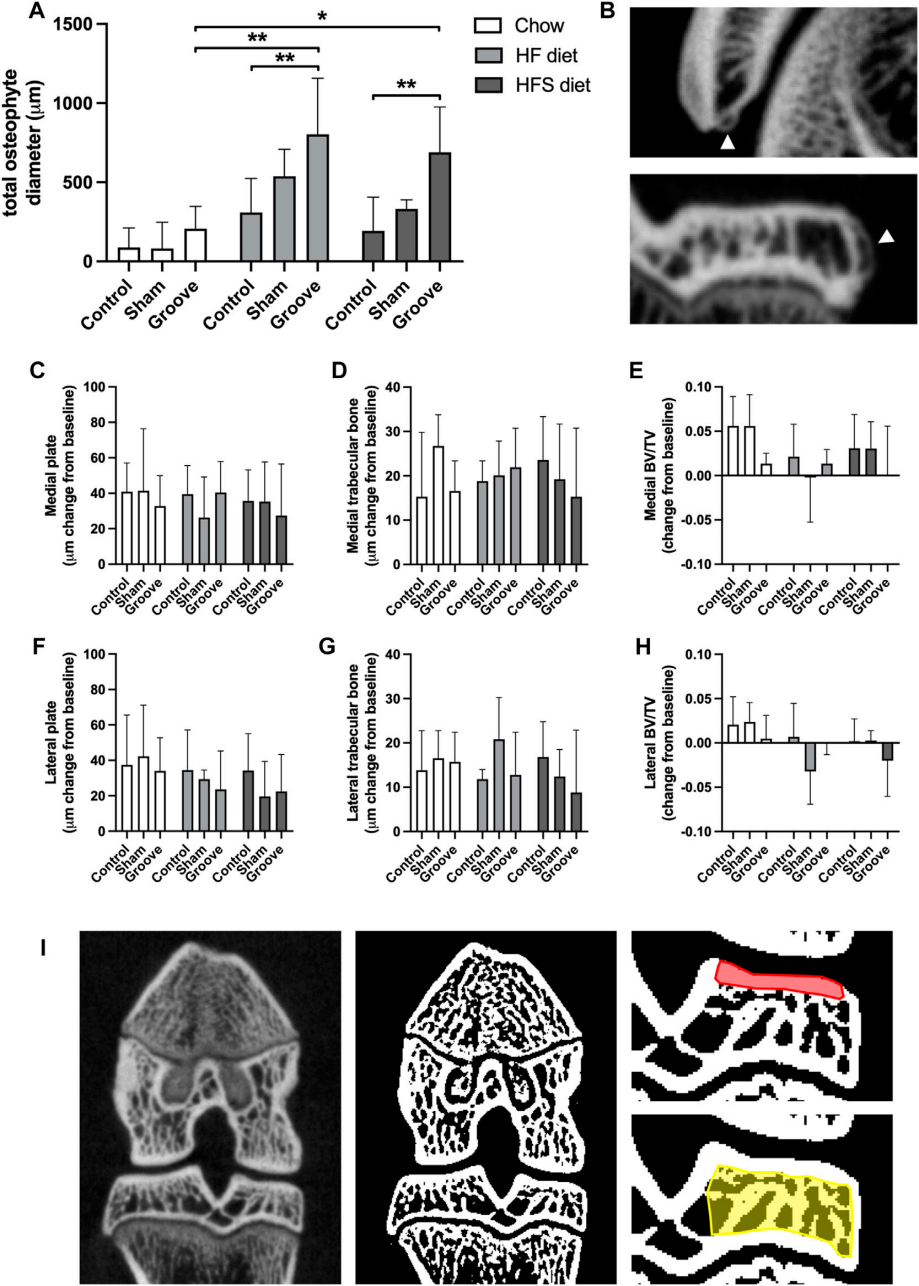
Effects of diet on micro-CT measurements **(A)** Total osteophyte diameter measured on micro-CT scan. **(B)** Images of osteophytes on the patella (upper) and tibia (lower), arrowheads indicate osteophytes. **(C–H)** Micro-CT measurements presented as change from baseline: **(C)** medial and **(F)** lateral tibial subchondral plate thickness, **(D)** medial and **(G)** lateral tibial trabecular thickness, **(E)** medial and **(H)** lateral tibial trabecular bone volumetric fraction. **(I)** CT image (left) and corresponding segmented bone (middle), in red the ROI for subchondral plate is shown in, and in yellow the ROI for trabecular measurements.

**FIGURE 5:**
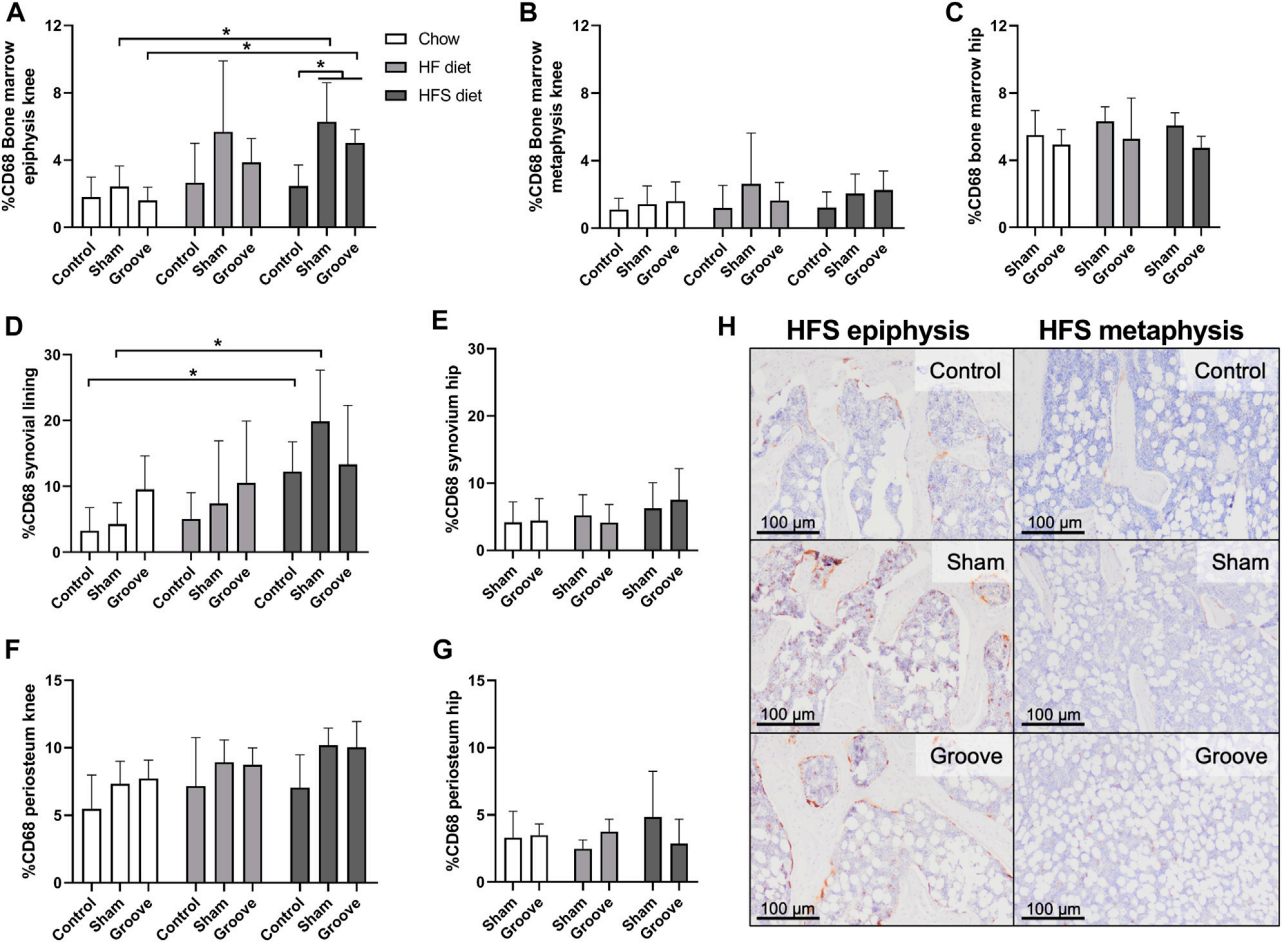
Effects of diet on CD68 monocyte/macrophage marker in knee and hip tissues. Percentage CD68 staining in the bone marrow of **(A)** knee tibia epiphysis, **(B)** knee tibia metaphysis, and **(C)** hip femur. Percentage CD68 staining in the synovial lining of **(D)** knee joints, and **(E)** hip joints. Percentage CD68 staining in the periosteum of the **(F)** knee tibia, and **(G)** hip femur. **(H)** Representative immunohistochemical images of CD68 staining in the knee tibia bone marrow of HFS fed animals. The difference in staining intensity between the epiphysis and the metaphysis of the same animal can be observed for a control, sham and grooved joint.

Sample size was determined *a priori* based on power analysis using G*power. We considered a difference between means of 3 points in the primary outcome (total OARSI score) clinically relevant, with a corrected α = 0.01, β = 0.2, and aiming for a power of 80%. Based on previous research the variance in grooved animals was estimated at σ^2^ = 1.21 and in ungrooved animals σ^2^ = 0.56 (de Visser et al., 2017; de Visser et al., 2018a; de Visser et al., 2019; Warmink et al., 2022; Warmink et al., 2023). This resulted in 4 animals in the grooved groups and 3 animals in the ungrooved groups, thus a total of 7 animals per diet group would be sufficient. To account for potential loss of animals among obese groove-operated animals (estimated risk 3%–5%), we included one extra animal in the HF and HFS grooved groups. Additionally, for animal welfare considerations, we added one more animal to the chow group to ensure an even number of animals that could be housed in pairs, with the same diet per cage. This additional rat received sham surgery to minimize discomfort. Statistical analysis was performed using Prism (v7.04, GraphPad Software, La Jolla, CA, United States) and IBM SPSS software (v25.0, IBM SPSS Inc., Chicago, IL, United States). Differences between diet groups were tested using one-way ANOVA using Sidak’s multiple comparisons test (for the data presented in Figures 1C–E, Figures 4, 5, Supplementary Figures S3, S4). Next to every ANOVA performed, a Q-Q plot of residuals was made and checked for data distribution. Data that did not follow a normal distribution (total OARSI score, OARSI subscores), were log-transformed to meet statistical requirements, and tested using one-way ANOVA using Sidak’s multiple comparisons test (Figure 3). This approach was preferred over a Kruskal-Wallis test, which would only indicate a potential difference in the distribution between the groups, with the current sample size. When comparing data within animals (left and right legs), differences were tested using a two-tailed paired *t*-test (for the data presented in Supplementary Figure S4). Longitudinal data (for the data presented in Figures 1A, B, Figure 2) was tested using a mixed model (REML) analysis, with Tukey’s correction for multiple comparisons. The time point and rat strain were set as fixed effects and the individual rat as random effect. *p*-values ≤0.05 were considered statistically significant. Graphs represent mean +/− standard deviation (SD), unless noted otherwise, *p*-values in graphs are reported with *, # or & where *p* ≤ 0.05 is marked by one symbol (*), *p* ≤ 0.01 is marked by two symbols (**), *p* ≤ 0.001 is marked by three symbols (***), *p* ≤ 0.0001 is marked by four symbols (****) and *p* > 0.05 is not significant (NS). Observers were always blinded with respect to group divisions while performing the measurements in a randomized order.

## Results

### HFS diet results in the largest metabolic dysregulation

After 24 weeks of diet intervention, body mass was highest in the HFS group and the lowest in the chow group, however differences between groups were not statistically significant (Figure 1A; *p* = 0.07). To quantify the metabolic syndrome development, insulin resistance (the HOMA-IR) was calculated. At baseline (week 0), the HOMA-IR was similar between the three diet groups (Figure 1B). At week 12, we observed a higher insulin resistance (HOMA-IR) in the HF and in the HFS groups compared to the chow group (*p* = 0.004 and *p* = 0.02, respectively), and no difference between HFS and HF groups (*p* = 0.62). In week 24, the chow group also had the lowest HOMA-IR, but at this time point we observed a larger variation and no statistically significant difference compared to HF and HFS groups (*p* = 0.40 and *p* = 0.06, respectively). CISI was determined as a measure for insulin sensitivity by glucose tolerance test at week 24. CISI was lower in the HFS group compared to chow group (Figure 1C; *p* = 0.04), while HF and chow groups did not differ (*p* = 0.51), indicating HFS diet had the largest negative effect on insulin sensitivity. Furthermore, serum leptin level was higher in the HFS group compared to the chow group (Figure 1D, *p* = 0.008) and not significantly different between chow and HF groups (*p* = 0.50) and between HF and HFS groups (*p* = 0.09). In addition, serum cytokines measured on multiplex showed a difference between groups in RANTES, with an increase in the HFS group compared to chow and HF groups (Figure 1E; *p* = 0.03 and *p* = 0.047, respectively). Other cytokines did not show any significant difference between groups (Supplementary Figure S3), but normalized visualization (for each cytokine highest value was set to 100, lowest value set to 0) on heat map did reveal some individual animals in the HFS group that had relatively high systemic levels of cytokines (Figure 1F).

### Both HF and HFS diets cause an increase in intermediate monocytes

To determine the effects of diet on blood monocyte subsets, flow cytometry was used (Figure 2A). Cells with lower granularity were first selected in a forward/side scatter plot, then monocytes were selected by gating CD172a^+^ cells and monocyte subsets were differentiated by CD43 expression (Supplementary Figure S1). The overall blood CD172a^+^ monocyte population (as a percentage of total white blood cells) increased over time between week 1 and 23 (Figure 2B, *p* = 2.9 × 10^−5^). At none of the time points we observed a difference between the diet groups. The monocyte percentage was at the highest level for all three groups in week 17, five weeks after surgery, suggesting the surgery procedure might have influenced the monocyte level. In addition, the percentage of CD172a^+^CD11b/c^+^ cells, comprising the monocyte and dendritic cell populations, was high at week 1, 6 and 11 in all groups (>80%), and dropped significantly after surgery in week 17 (Figure 2C; *p* = 3.9 × 10^−8^, *p* = 6.0 × 10^−10^ and *p* = 6.2 × 10^−9^, respectively), subsequently remaining lower in week 23 (*p* = 1.0 × 10^−7^, *p* = 7.0 × 10^−10^ and *p* = 1.8 × 10^−8^, respectively), but no difference was observed between diet groups (*p* = 0.55).

The total monocyte population was further differentiated into CD172a^+^CD43^low^ classical, CD172a^+^CD43^int^ intermediate and CD172a^+^CD43^hi^ non-classical monocytes (Figure 2D). The CD172a^+^CD43^int^ intermediate monocyte population showed the largest increase in response to HF and HFS diets compared to the chow diet, starting at week 11 (Figure 2E; *p* = 0.003 and *p* = 0.002, respectively) and continuing at week 17 (*p* = 2.1 × 10^−5^ and *p* = 0.001, respectively) and at week 23 (*p* = 0.001 and *p* = 0.005). Indicating that both HF and HFS diets cause an increase in CD172a^+^CD43^int^ intermediate monocytes. Classical monocytes in the HF and HFS groups were significantly higher at end point (week 23) compared to the start at week 1 (Figure 2F; *p* = 0.01 and *p* = 0.02, respectively), whereas the chow group showed no significant increase (*p* = 0.1), indicating that the classical monocytes are affected by the HF and HFS diets over time. In addition, comparisons between diet groups per time point showed that in the HF group the percentage of classical monocytes was higher compared to chow at week 6 (*p* = 0.005), and higher in the HFS groups compared to chow at week 23 (*p* = 0.04). At all other time points a similar trend was observed, where the chow group had the lowest level of classical monocytes, but not statistically significant. In the non-classical monocyte population, the opposite trend was observed, where the chow group always had the highest percentage (Figure 2G). In the non-classical populations, the HF and HFS groups decreased slightly between week 1 and 23 (*p* = 0.03 and *p* = 0.004, respectively) and the chow group remained similar between week 1 and 23 (*p* = 0.1). The non-classical monocyte population was lower in the HF group compared to chow at week 6 (*p* = 0.004), both HF and HFS groups were lower compared to chow at week 17 (*p* = 0.01, and *p* = 0.01, respectively), and at week 23 the HFS group was lower compared to chow (*p* = 0.008). So, the intermediate monocytes showed the clearest effect in response to HF and HFS diets. Even though classical and non-classical monocyte levels show more fluctuation over time than intermediate monocytes, we do observe a clear trend where classical monocytes increase in response to HF and HFS diets and non-classical monocytes decrease.

### HFS diet increases susceptibility for knee joint damage after minimal surgical intervention

Left non-surgical control joints showed similar results for all the measured histological outcomes, independent from groove or sham surgery in the animals’ contralateral (right) joint. Indicating groove or sham surgery in the right joint did not affect OA, osteophyte or CD68 levels in the contralateral left joint. Therefore, all control left joints are treated as one group in Figures 3–5, and in their statistical analysis. The same data with separate control groups and separate statistical analysis are shown in Supplementary Figure S4.

On knee joint integrity, we observed an increase in the total OARSI score in the grooved knee of HF and HFS group compared to control knee (Figure 3A; *p* = 0.02 and *p* = 0.004, respectively, statistics using log-transformed data) (Supplementary Figure S5). In addition, we found that HFS sham group had an increased total OARSI score when compared to HFS control (*p* = 0.0003, statistics using log-transformed data), and when compared to chow and HF sham groups (*p* = 0.0014 and *p* = 0.0035, respectively, statistics using log-transformed data). This suggests that the minimal damage induced by sham surgery was enough for the HFS fed animals to develop joint damage to a similar extent as the grooved joints in HF and HFS groups. The differences in total OARSI score were mainly caused by differences in the OARSI cartilage degeneration subscore (Figures 3B, C), as the subchondral bone, synovium and osteophyte subscores showed no differences between groups (Figures 3D–G). However, when osteophyte size was quantified on micro-CT, we observed an increase in total osteophyte diameter in the HF groove and HFS groove groups compared to HF and HFS control (Figures 4A, B; *p* = 0.004 and *p* = 0.004, respectively) and compared to the chow groove group (*p* = 0.003 and *p* = 0.03, respectively). Osteophyte diameter was small in all chow fed rats relative to HF and HFS fed rats. In addition, micro-CT measurements of medial and lateral subchondral plate thickness, trabecular bone thickness and bone volume fraction showed no differences between groups (Figures 4C–I).

### In bone marrow epiphysis, local CD68 monocyte/macrophage marker increases only when obesogenic diet and surgical injury are combined

The percentage of CD68 staining was measured in bone marrow, synovial lining and the periosteum to determine effects of diet and OA induction on presence of monocyte and macrophages in knee and hip joints. In the bone marrow of the epiphysis of the tibia we observed an increase in CD68 staining in HFS sham and HFS groove compared to HFS control (Figures 5A, H; *p* = 0.04 and *p* = 0.05, respectively). CD68 staining in the HFS sham and HFS groove groups were also increased compared to the chow sham and grooved groups (*p* = 0.04 and *p* = 0.04, respectively). This indicates that the combination of HFS diet and surgical intervention by either groove or sham surgery increases CD68 staining in the bone marrow of the tibial epiphysis thus matching the findings of the OARSI scores in Figure 3. HFS diet alone did not cause an increase in CD68 staining as indicated by the contralateral control joints. The HF sham and HF grooved group showed a similar trend, but was not statistically significantly increased from the chow groups (*p* = 0.06 and *p* = 0.1, respectively). The CD68 staining in the bone marrow of the metaphysis was measured on the same histological section as the epiphysis, and in the metaphysis we did not detect any effect of diet or surgical intervention on the percentage of CD68 staining (Figure 5B). In addition, CD68 staining in the hip bone marrow was not affected by diet or knee joint surgery (Figure 5C), indicating that the increase in CD68 in the tibial epiphysis bone marrow is a very localized response. In the knee joint synovial lining we observed an increase in CD68 staining in the HFS control and HFS sham group compared to the chow control and chow sham group (Figure 5D; *p* = 0.04 and *p* = 0.03, respectively). Indicating we see some increase in macrophages in the synovial lining as a result from HFS diet. The CD68 staining in the synovium of the hip was not affected by diet and showed no difference between groups (Figure 5E). In the periosteum of the tibia and hip we saw no difference between diet groups (Figures 5F, G). The sham and grooved groups always had the highest level of periosteum CD68 staining, but this was not statistically significantly different from their respective control groups (*p* > 0.5).

## Discussion

Our data shows that rats fed with HF and HFS diets develop comparable levels of joint damage in response to groove surgery, and that both diets result in more joint damage than a regular chow diet. Interestingly, in rats fed a HFS diet, even a minimal disturbance of the knee through sham surgery seems sufficient to trigger joint damage, which was not observed in HF or chow fed animals. The joint damage observed in the HFS sham group (and perhaps, in part, also in the grooved groups) is most likely related to tissue damage caused during the sham surgery, which involved a longitudinal incision the patellar tendon, piercing the infrapatellar fat pad and synovium, and possible injury-related bleedings (de Visser et al., 2017). Other studies report variations in pain behavior and weight-bearing asymmetry after sham surgery in mouse DMM models, indicating that sham joints cannot be considered naïve (Gowler et al., 2020). Furthermore, DMM and sham surgery induce similar inflammatory responses in synovial thickness and synovial macrophages, compared to naïve joints (Utomo et al., 2021). However, these changes in the sham group did not lead to cartilage damage, in contrast to DMM mice (Utomo et al., 2021). In addition, a critical review of the literature regarding sham surgery in clinical trials, has highlighted that several confounding factors can impact the reliability of sham surgery (Ciccozzi et al., 2016). Thus, this can be considered a limitation of all *in vivo* research using sham surgery as control group, as it raises concerns about the validity for hypothesis testing. Nevertheless, despite these limitations, we observe a sham-induced effect exclusively in the HFS group, making it a unique observation that could help provide insight into the pathogenesis of metabolic OA. It is reported that immune cells, and especially macrophages, located in joint tissues will activate in response to tissue damage and subsequently secrete inflammatory mediators (Scanzello et al., 2008; Wynn and Vannella, 2016). In addition, blood release into the joint cavity, for instance by bloody joint effusion or hemarthrosis, increases the risk of OA (Gelber et al., 2000; Potpally et al., 2021). The immune cells, erythrocytes, and other blood components directly impact cartilage matrix integrity and chondrocyte health (Roosendaal et al., 1997), and can induce an inflammatory reaction in the synovium (Madhok et al., 1991; Roosendaal et al., 1998; Ovlisen et al., 2009). These factors collectively contribute to catabolic changes within the joint, leading to OA development, and possibly explaining the joint damage observed in the HFS sham group. Next to joint damage, we observed that HFS diet leads to the largest metabolic disturbance by HOMA-IR, CISI, and leptin, as well as the highest (CD68^+^) synovial inflammation, which could explain the increased susceptibility to develop joint damage specifically in the HFS sham group. Previous studies have demonstrated increased metabolic dysregulation in mice exposed to diets high in both carbohydrates and fat (Tordoff and Ellis, 2022), and in humans high sugar intake is associated with an elevated risk of metabolic syndrome (Malik et al., 2010; Rodriguez et al., 2016). The effects of the HFS diet on synovial inflammation and macrophage CD68 expression have been relatively understudied. However, studies have also shown a connection between high-fat high-carbohydrate diets and increased synovial inflammation and macrophage infiltration in animal models (Larranaga-Vera et al., 2017; Sun et al., 2017). These are very relevant findings, linking metabolic dysregulation and inflammation to metabolic OA development.

Monocyte subsets are known to be affected by obesity and are implicated in many inflammatory diseases. Here, we observed that both obesogenic diets resulted in a shift from non-classical towards classical monocytes with a marked increase in intermediate monocytes, suggesting higher cellular inflammation levels compared to chow fed rats. This agrees with previous findings where obesity was shown to increase the intermediate monocyte subset in humans (Poitou et al., 2011). Intermediate monocytes can secrete high levels of inflammatory cytokines, and in patients with OA are associated with lower functional scores (Wong et al., 2012; Gomez-Aristizabal et al., 2019). In rats, the markers CD172a and CD43 are typically used to select for monocytes, where CD172a^+^CD43^hi^ monocytes seem to be analogous to murine Ly6C^low^ and human CD14^+^CD16^+^ non-classical monocytes, and CD172a^+^CD43^low^ monocytes seem to be analogous to murine Ly6C^hi^ and human CD14^hi^CD16^-^ classical monocytes (Strauss-Ayali et al., 2007; Melgert et al., 2012; Zhou et al., 2013; Barnett-Vanes et al., 2016). Here, not only high and low CD43-expressing monocytes were differentiated (Ahuja et al., 1995), but also intermediate CD43 expressing monocytes, that showed a CD43 expression state in between classical and non-classical monocytes. We observed a high level of non-classical monocytes (60%–90%) and a low level of classical monocytes (5%–25%), which is different from human where the opposite pattern is observed, with high levels of classical monocytes and low levels of non-classical monocytes, and mouse, where the populations are approximately evenly distributed (Strauss-Ayali et al., 2007). The percentage of intermediate monocytes seems to be low in all species, as we show in this study as well. We observed an increase in classical monocytes over time for the HF and HFS diet group, accompanied by a decrease in non-classical monocytes. This contradicts findings in humans with obesity and inflammatory diseases, where an elevation in non-classical monocytes is commonly observed (Cottam et al., 2002; Poitou et al., 2011; Wong et al., 2012; Pecht et al., 2016), and an elevation in classical monocytes is rarely observed (Schipper et al., 2012; Friedrich et al., 2019). In addition, the latter study only found a difference in non-classical monocytes after stratifying by type 2 diabetes mellitus disease status (Friedrich et al., 2019), suggesting that comorbidities, such as type 2 diabetes mellitus, may act as confounding factors in the relationship between obesity and monocyte subpopulations. In mouse models of obesity, conflicting results have been reported, with some studies demonstrating a decrease in classical monocytes and others showing an increase, similar to our observations (Tacke et al., 2007; Liu et al., 2020). Part of these discrepancies might be explained by the variation in time points investigated (Tacke et al., 2007). Notably, in a mouse model of rheumatoid arthritis, non-classical monocytes have been implicated in driving the disease (Misharin et al., 2014). Interestingly, both obesogenic diets used in our study led to a significant increase in intermediate monocytes, starting from week 11, which occurred prior to the OA-inducing surgery. This finding aligns with observations in humans, where elevated levels of intermediate monocytes have been reported in individuals with obesity, as well as in rheumatoid arthritis and OA synovial fluid (Wong et al., 2012; Friedrich et al., 2019; Gomez-Aristizabal et al., 2019).

In addition, we determined the level of CD11bc^+^ cells, CD11b is a component of complement receptor 3 and CD11c of complement receptor 4, both found to be more abundant on the cell surface when monocytes are activated (Kiefer et al., 2021). In mice, it was shown that CD11b^+^ monocytes increase in response to diet induced obesity (Takahashi et al., 2003). A limitation of the marker used in this study is that both CD11b and CD11c are recognized by the antibody, as they have a shared epitope, and no CD11b or CD11c antibodies were available for rat in a suitable combination for our flow cytometry panel. The simultaneous CD11b and CD11c recognition could explain why we observed that the CD11bc expression was high (>80%) at baseline and no increase in CD11bc expression could be observed. In humans, CD11b expression is known to increase depending on the monocyte maturation stage (Lambert et al., 2017), therefore an increase in total monocytes population with less matured monocytes and lower CD11b expression could cause a decrease in overall CD11b expression, although the CD11b expression for rat monocyte maturation stages are unknown. In addition, non-classical monocytes in the rat are found to have higher expression of CD11c (Barnett-Vanes et al., 2016), therefore the decrease we observed in non-classical monocytes over time could also have contributed to the decrease in CD11bc expression. Together, these factors may have overruled any effects of diet and/or joint damage on CD11bc monocyte activation, as we observed no differences between groups.

Interestingly sham surgery in combination with obesogenic diet also led to an increase of CD68 in the bone marrow, which was most pronounced in HFS animals. This effect was exclusively observed in the bone marrow of the groove/sham tibia epiphysis, and not in the tibia metaphysis or other (knee and hip) joints of the same animal, suggesting a local effect related to joint damage. However, no high levels of joint damage or CD68 inflammation occurred in any of the chow fed groups. Thus, it appears that the combination of an obesogenic diet and joint damage, is needed in this Wistar rat model for CD68 positive cells as well as OA development, as observed in previous studies (de Visser et al., 2018b; Warmink et al., 2020). In addition, we did not observe any damage nor CD68 positive cells in left control joints correlated to high joint damage in the animals’ right joint, indicating OA and CD68 expression in one knee joint does not affect joints without surgical damage in this model, independent of the diet. In contrast, other models are known to develop joint damage in response to an obesity-inducing diet alone (Kozijn et al., 2018; Rios et al., 2019). In light of our results, we hypothesize that the increased metabolic loading caused by an obesogenic diet, will prime monocytes and macrophages in the blood, bone marrow and joint tissues. When subsequently triggered by damage in the joint, it results in OA development. However, when the effect of the diet on the metabolic loading is larger, such as we observed here for HFS diet fed rats, the surgical trigger that is needed seems to be smaller, or even completely redundant as observed in other models (Collins et al., 2018; Kozijn et al., 2018; Rios et al., 2019). So, while the current study provides evidence that monocyte and macrophage activation may play an important role in the systemic component of OA development, it was not sufficient to induce joint damage by itself in our obesogenic rat model.

In addition to indirect effects of diet on OA development, via metabolic dysregulation and low-grade inflammation, dietary fats and sugars are also known to exert direct, and independent, effects on chondrocyte metabolism. A mouse study using purified diets, showed that high-fat content seems to increase cartilage lipid metabolism, while a high sucrose diet causes a modest increase in carbohydrate pathways (Donovan et al., 2018). Chondrocytes store substantial amount of lipids, and increased systemic cholesterol is known to affect chondrocyte lipid metabolism (Iliopoulos et al., 2008; Gkretsi et al., 2011). *In vitro*, exposure of chondrocytes to single fatty acids leads to different effects depending on the type of fatty acid. Omega-3 fatty acids cause lower expression of inflammatory mediators (Curtis et al., 2002), whereas linoleic acid (omega-6 fatty acid) decreases collagen synthesis and lipid peroxidation in cartilage matrix increases collagen degradation (Watkins et al., 1996; Tiku et al., 2000). In addition, bone of OA patients contains high levels of fat and omega-6 fatty acids (Plumb and Aspden, 2004). Dietary sugars also affect chondrocyte health. It was shown that chondrocytes cultured in high glucose medium increase their polyol methabolic pathway resulting in enhanced chondrocyte inflammation (Laiguillon et al., 2015; Donovan et al., 2018). OA cartilage explants from diabetes mellitus patients had an increased responsiveness to Il-1B induced inflammation, compared to age and BMI matched non-diabetic controls (Laiguillon et al., 2015). In women, both increased systemic cholesterol and blood glucose levels are associated with knee OA, independent from obesity (Hart et al., 1995). Aditionally, elevated blood glucose is associated with joint space narrowing in knee OA, after adjustment for BMI (Eymard et al., 2015). Thus, both elevated sucrose and fat intake can contribute to the development of OA independent from the effects of diet on weight or metabolic disturbance. In our study we cannot draw conclusions on the independent effects of dietary fat and sucrose, our HF diet contained no sucrose, whilst the HFS diet contained high amounts of sucrose on top of a high-fat content.

## Conclusion

In conclusion, we find that two obesogenic diets in combination with groove surgery result in similar joint damage in the Wistar rat, and minimal sham damage was enough to trigger joint damage in the HFS fed rats. We suspect this might be explained by the increased metabolic disturbance that was observed in the HFS group, resulting in a smaller trigger needed to incite the processes of joint degeneration. Both obesogenic diets cause a shift in monocyte populations resulting in an increase in intermediate monocytes as well as increased CD68 expression in the bone marrow and synovial lining. These findings are of importance to understand the factors that contribute to development of the metabolic OA phenotype in human patients, as their underlying etiology might differ from other patients with OA and may, therefore, need different diagnostic and therapeutic approaches.

## Data Availability

The raw data supporting the conclusion of this article will be made available by the authors, without undue reservation.

## Supplementary material

SUPPLEMENTARY FIGURE S1 Flow cytometry gating strategy. First, small debris was deselected in an SSC-A/FSC-A plot, this gate was called “no debris”. Then, singlets were selected from the “no debris” population in an FSC-H/FSC-A plot. From the singlets population, live cells were selected using DAPI staining. Next, from the live cells population, cells with a relatively low level of granulation were selected in a SSC-A/FSC-A plot, these were called “mono- and lymphocytes”. From the “mono- and lymphocytes” population CD172a+ cells were selected. From the CD172a+ cells, both total CD11bc+ population and three monocyte subsets were determined by CD43 low, intermediate and high expression.

SUPPLEMENTARY FIGURE S2 Methods of digital CD68 quantification. The region of interest (ROI) was manually drawn in each image. The region of interest included all areas with the structure of interest (bone marrow, synovial lining or periosteum) that was observed in the image. Images were deconvoluted and the brown colour-separated image was used to determine the percentage area of positive staining using threshold (shown in red) and measure functions.

SUPPLEMENTARY FIGURE S3 Week 24 blood serum cytokine levels in chow, HF and HFS groups. No differences between groups are observed.

SUPPLEMENTARY FIGURE S4
**(A)** Osteophyte, **(B)** OARSI and **(C–F)** CD68 data showing two (unpooled) control groups. Data with pooled controls is presented in Figures 3, 4.
