# Supplementary figures and images for: Effects of different obesogenic diets on joint integrity, inflammation and intermediate monocyte levels in a rat groove model of osteoarthritis
Source: Front Physiol. 2023 Jul 13;14:1211972. doi: 10.3389/fphys.2023.1211972 (PMC10372350; doi:10.3389/fphys.2023.1211972)

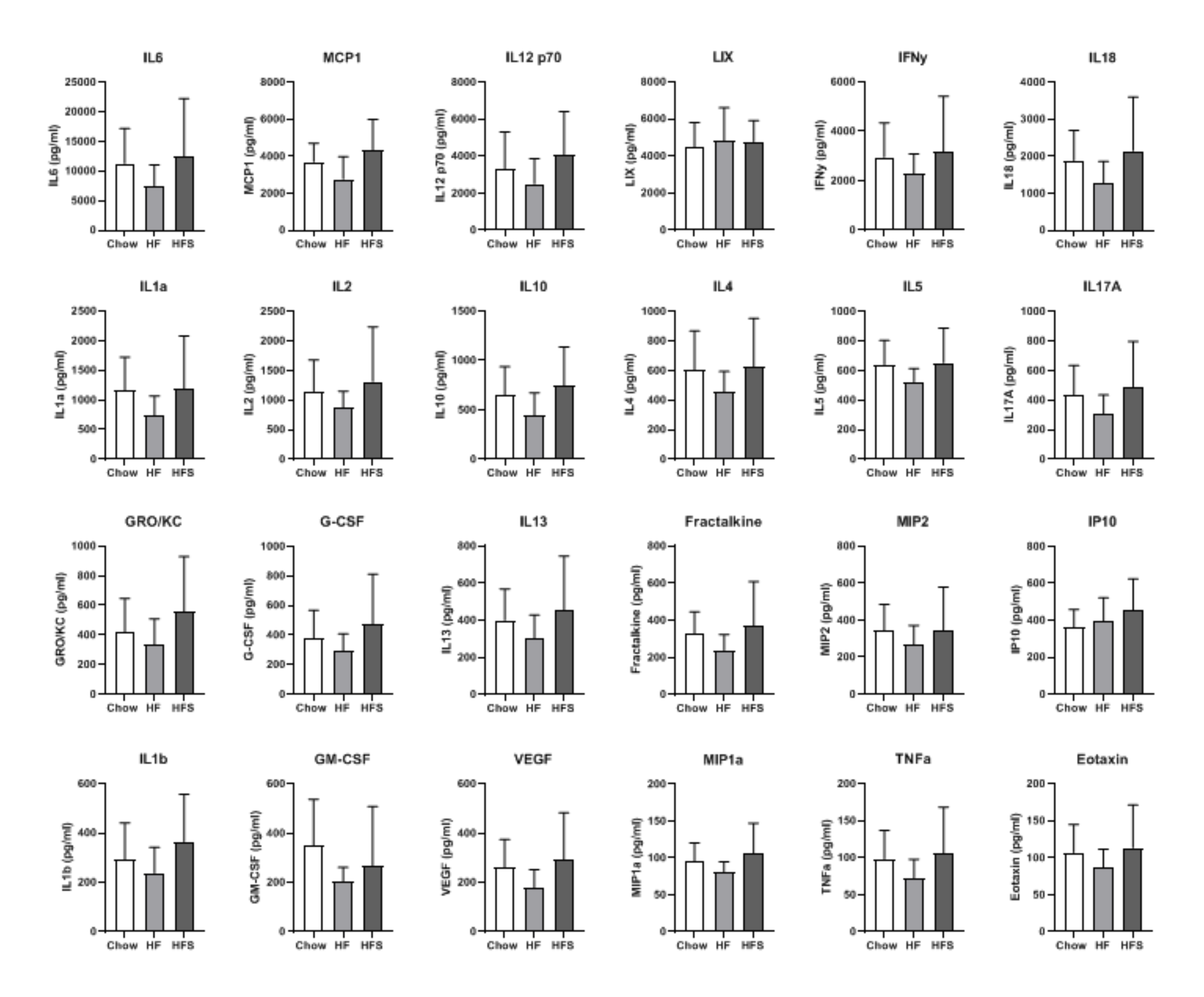

Supplement: Supplementary file 1 [file Image3.tif]

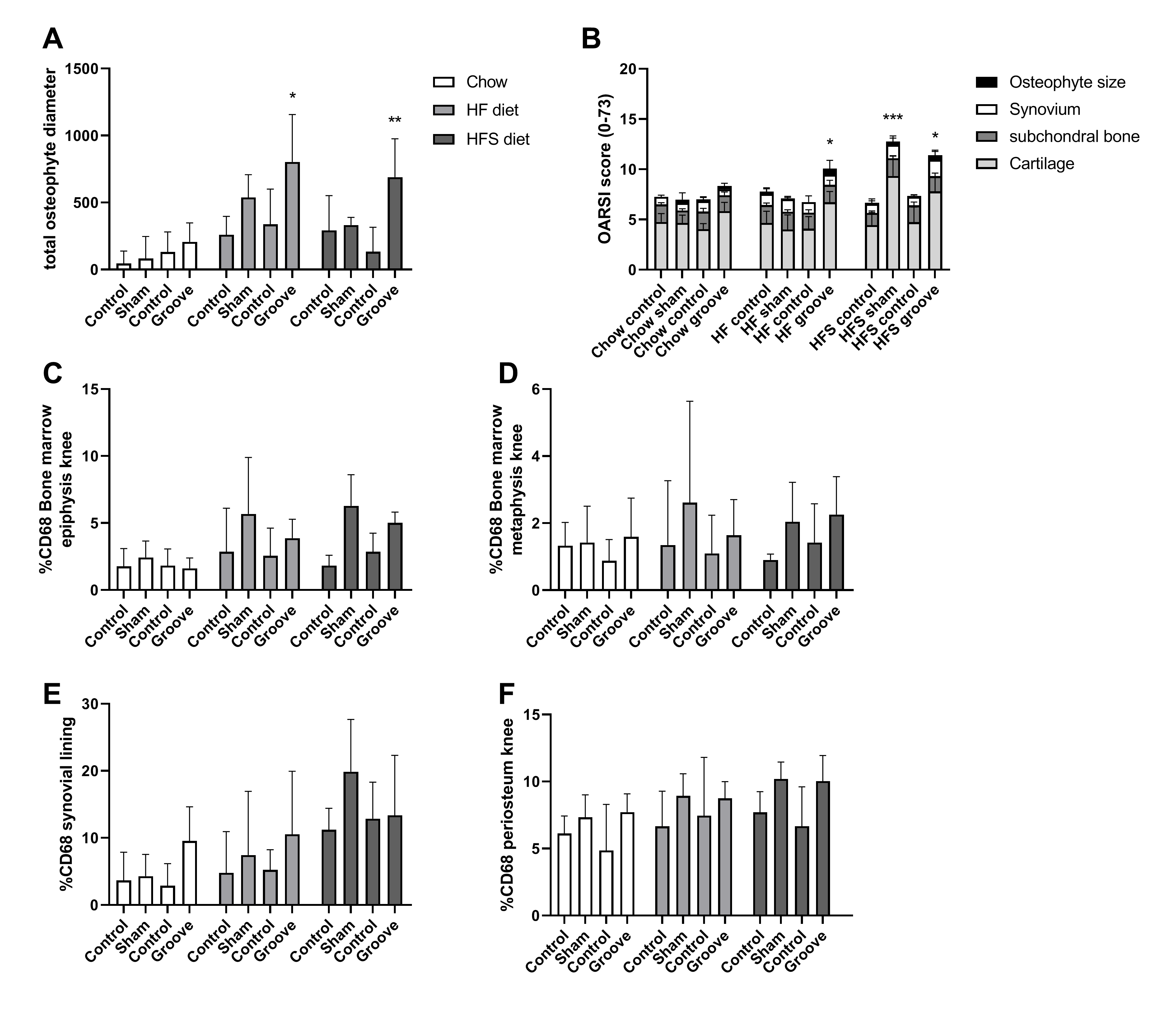

Supplement: Supplementary file 2 [file Image4.tif]

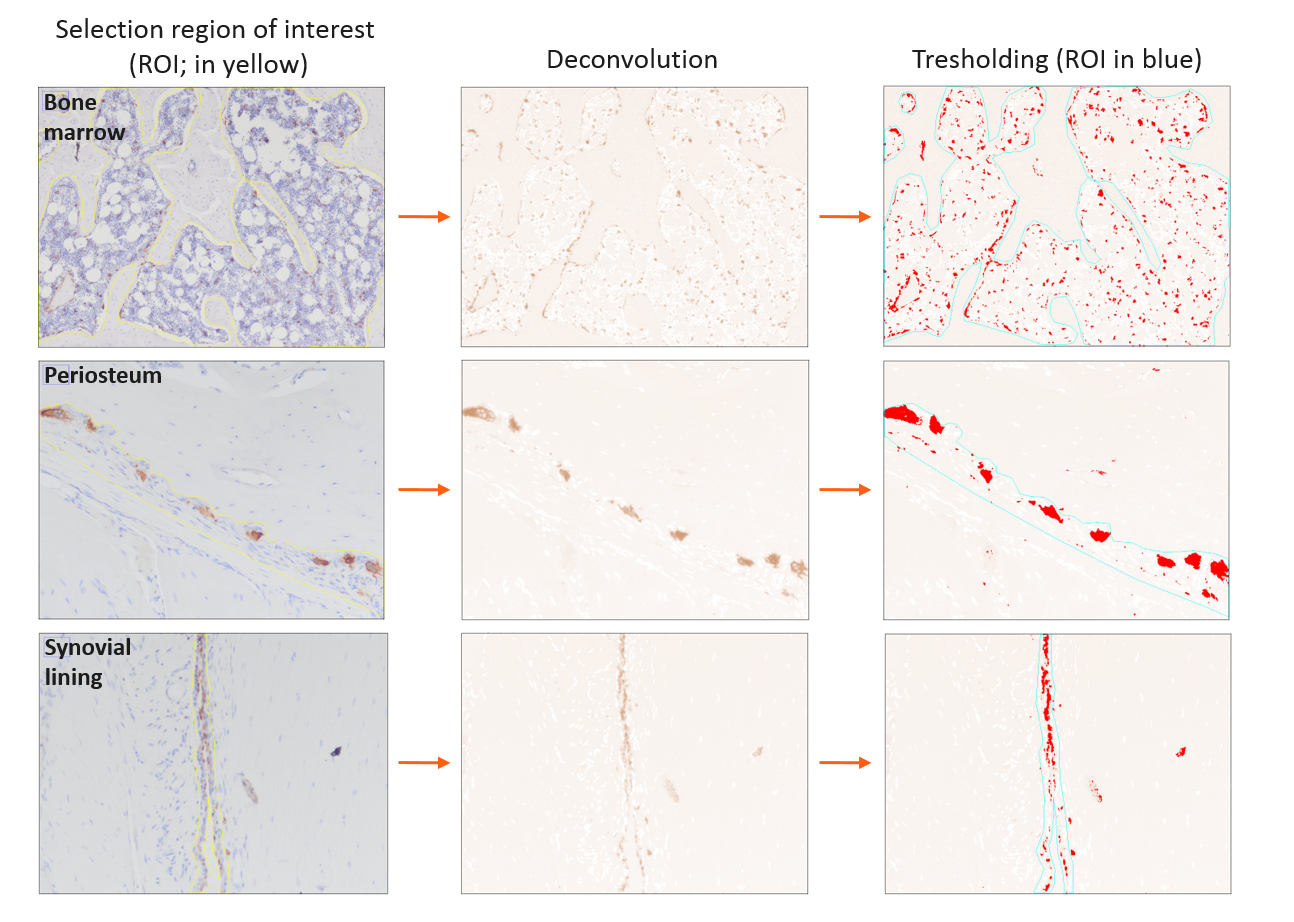

Supplement: Supplementary file 3 [file Image2.tif]

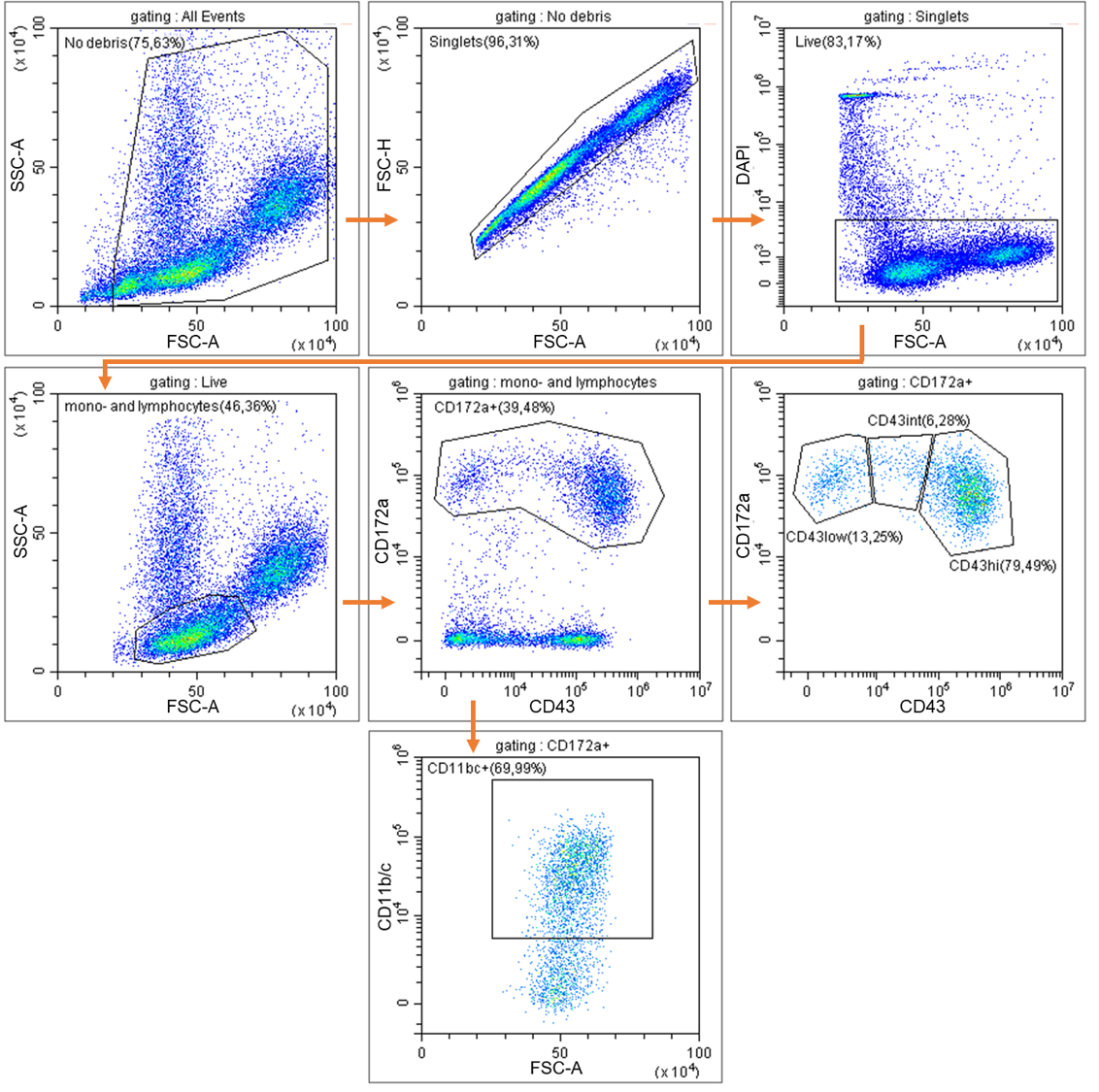

Supplement: Supplementary file 4 [file Image1.tif]
